# The EF-hand calcium-binding protein tescalcin is a potential oncotarget in colorectal cancer

**DOI:** 10.18632/oncotarget.1851

**Published:** 2014-03-19

**Authors:** Yun Hee Kang, Seung Ro Han, Jong-Tae Kim, Seon-Jin Lee, Young Il Yeom, Jeong-Ki Min, Chul-Ho Lee, Jae Wha Kim, Suk Ran Yoon, Do-Young Yoon, Kwan Soo Hong, Geum-Sook Hwang, Hee Cheol Kim, Young-Ha Lee, Hee Gu Lee

**Affiliations:** ^1^ Medical Genomics Research Center, Korea Research Institute of Bioscience and Biotechnology, Daejeon, Korea,; ^2^ Department of Biological Science, Daejeon University, Daejeon, Korea,; ^3^ Department of Bioscience and Biotechnology, BIMC, Konkuk University, Seoul, Korea,; ^4^ Seoul Center, Korea Basic Science Institute, Seoul, Korea,; ^5^ Department of Surgery, Samsung Medical Center, Sungkyunkwan University School of Medicine, Seoul, Korea,; ^6^ Department of Infection Biology, Chungnam National University School of Medicine, Daejeon, Korea

**Keywords:** Tescalcin, colorectal cancer, cell growth, tumor growth, NF-κB

## Abstract

Tescalcin (TESC) is an EF-hand calcium binding protein that is differentially expressed in several tissues, however it is not reported that the expression and functional roles of TESC in colorectal cancer. Levels of messenger RNA (mRNA) and protein expression of TESC in colorectal cancer tissues were assessed using RT-PCR, real time PCR, immunohistochemistry, and clinicopathologic analyses. Quantitative analysis of TESC levels in serum specimens was performed using sandwich ELISA. Colorectal cancer cells transfected with TESC small interfering RNA and short hairpin RNA were examined in cell proliferation assays, phospho-MAPK array, and mouse xenograft models. Here we demonstrated that TESC is overexpressed in colorectal cancer (CRC), but was not expressed in normal mucosa and premalignant dysplastic lesions. Furthermore, serum TESC levels were elevated in patients with CRC. Knockdown of TESC inhibited the Akt-dependent NF-κB pathway and decreased cell survival *in vitro*. Depletion of TESC reduced tumor growth in a CRC xenograft model. Thus, TESC is a potential diagnostic marker and oncotarget in colorectal cancer.

## INTRODUCTION

Colorectal cancer (CRC) is one of the leading causes of cancer-related death worldwide. In the United States, CRC is the third most common form of cancer and the third leading cause of cancer-related death in both men and women [1-2]. The high mortality rate of patients with CRC can be attributed to the high risk of metastasis of this cancer. Despite the availability of several treatment regimens for metastatic colorectal cancer, many patients with CRC die as a result of metastatic spread within a few years of diagnosis [3-4]. Aggressive metastatic cancers are characterized by their high capacity for migration and subsequent invasion and adhesion in distant organs [5]. Acquisition of these properties by cancer cells involves specific changes in the expression level of several genes at the transcriptional and translational level.

NF-κB is a protein complex that controls the transcription of multiple target genes and plays a role in the regulation of immune responses and in cellular responses to diverse stimuli. Dysregulation of NF-κB signaling contributes to cancer cell proliferation, migration, and tumorigenesis [6-7]. Furthermore, activation of NF-κB is involved in the inflammatory responses associated with carcinogenesis [8]. NF-κB signaling is related to invasion and angiogenesis in colorectal cancer [9] and prevents apoptosis in CRC cells through JNK signaling and increased expression of antioxidant enzymes [10]. The well-established role of NF-κB in tumorigenesis suggests its potential as a target for anticancer therapies. Indeed, recent studies have shown that inhibition of NF-κB suppresses cell survival and tumor growth in head and neck squamous cell carcinoma [11].

*TESC* is an autosomal gene that is differentially expressed in mammalian tissues, with high expression in embryonic gonads [12], in adult mouse heart, brain, stomach, and testis, and in mouse and human primary hematopoietic progenitor cells and cell lines [13-14]. The 24-kDa TESC protein possesses a single functional EF-hand domain that binds Ca^2+^ with micromolar affinity [14-15]. Studies have shown that TESC regulates cellular pH by controlling activity of the plasma membrane Na^+^/H^+^ exchanger [16-19] and suppresses the phosphatase activity of calcineurin A *in vitro* [14]. Expression of TESC is effectively increased in response to the continuous activation of ERK1/2 during differentiation and maturation of megakaryocytes, when it plays a vital role in coupling the MEK/ERK cascade to expression of the Ets family of transcription factors [13].

In the present study, we showed that TESC was highly expressed in CRC tissues and serum compared with normal tissues. Further data indicated that TESC might regulate the proliferative activity of CRC cells *via* the Akt-dependent NF-κB signaling pathway. Our findings support a potential role of TESC as a diagnostic marker and an oncotarget in CRC.

## RESULTS

### Up-regulation of TESC mRNA and protein expression in colorectal cancer tissues and sera

**Figure 1:**
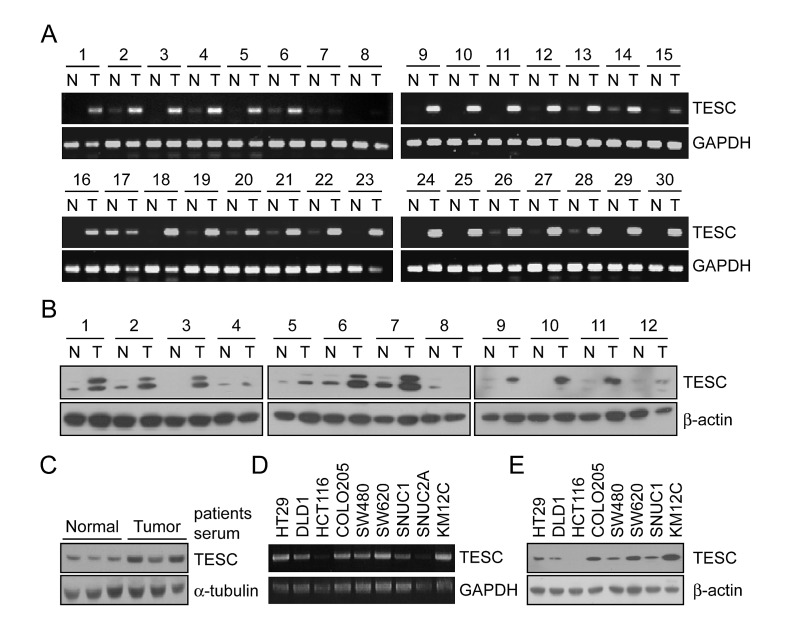
Increased mRNA and protein expression of TESC in human colorectal tissues, serum, and various CRC cell lines (A) RT-PCR analysis of TESC expression in 30 paired samples of non-tumor colon (N) and colorectal cancer (T) tissues. GAPDH was used as the loading control. (B, C) Western blot analysis of TESC expression in 12 pairs of non-tumor (N) and tumor (T) tissues (B), and in sera from patients with CRC (C). β-actin or α-tubulin was used as the loading control. (D, E) TESC expression in 10 CRC cell lines by RT-PCR (D) and Western blot (E) analysis.

To identify genes associated with the progression of CRC we analyzed gene expression profiles in paired tumor and adjacent non-tumor tissues from 66 patients with CRC using Illumina microarray chips. The expression of 281 genes was increased more than 2-fold compared with normal colorectal mucosa in at least 60% of CRC tissues. Cellular component ontology analysis was performed to identify genes located in the extracellular matrix that might be secreted into the serum of CRC patients. Among these genes (Supplementary Table 1), the expression of *TESC* was up-regulated more than 2-fold in 59 of the 66 cases (*P*=8.75e-17). To confirm results of DNA chip analysis, we analyzed *TESC* mRNA expression in 30 paired CRC tissue samples by RT-PCR and showed that transcriptional expression of TESC was significantly increased in all of the CRC tissues compared with the matched normal samples (Fig. 1A). Quantitative real time PCR showed that *TESC* mRNA levels were approximately 5 times (5.98 ± 7.26) higher in tumor tissues than normal tissues (n = 20) (Supplementary Fig. 1 and Supplementary Table 2). We also examined the translational expression of TESC in tissue and serum from control and colorectal cancer patients by Western blot analysis and showed that TESC protein levels were markedly increased in tissues and sera of patients with colorectal cancer (Fig. 1B, C). Finally, TESC mRNA and protein were differentially expressed in various colorectal cancer cell lines including HT29, DLD1, HCT116, COLO205, SW480, SW620, SNUC1, and KM12C (Fig. 1D). Interestingly, TESC protein was highly expressed in COLO205, SW480, SW620, and KM12C cell lines and moderately expressed in HT-29, DLD1, and SNUC1 cells, but TESC expression was not detected in the HCT116 cell line (Fig. 1E).

### Increased TESC expression is associated with poor prognosis in colorectal cancer patients

**Figure 2:**
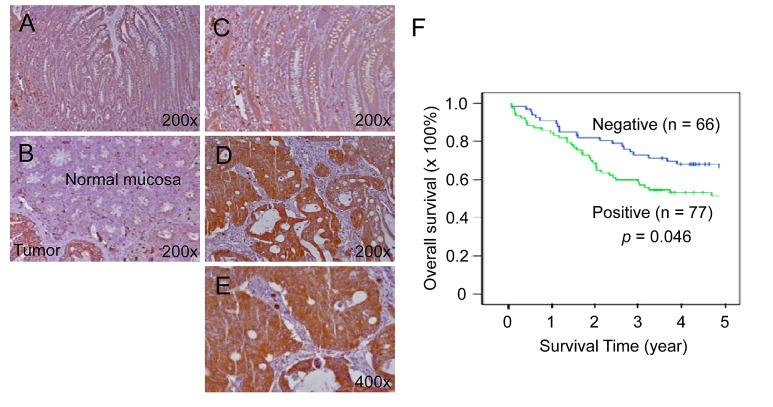
Increased expression of TESC in colorectal cancer tissues compared with normal tissues (A-E) TESC showed high expression in the cytoplasm of tumor cells of CRC tissues compared with normal colonic mucosal epithelium. TESC staining was low or negative in normal colonic mucosa (A, B) and dysplastic lesions (C), but high in colorectal cancer (D, E). (F) Kaplan-Meier survival curve according to TESC expression status. A significant difference in cumulative overall survival was observed between patients who were positive or negative for TESC expression.

We next evaluated TESC as a potential candidate tumor marker. TESC protein was strongly expressed in cancerous lesions of colorectal cancer tissues but slightly in normal mucosal epithelial cells by immunohistochemical analysis (IHC; Fig. 2A-E). In contrast, premalignant dysplastic lesions (20 tubular and tubulovillous adenomas with dysplasia) showed slight expression of TESC protein (data not shown). The staining intensity of TESC was weak or negative in normal colonic mucosa (Fig. 2A-C), but adenocarcinomas showed strong TESC expression (Fig. 2D, E). In particular, TESC was strongly expressed in the cytoplasm of tumor cells from CRC tissues compared with normal colonic mucosa and was also intensely expressed on the luminal side of the CRC glandular structures (Fig. 2E). Table 1 shows associations between TESC expression and clinicopathologic characteristics. Comparison of patients with high TESC expression and those with negative or low TESC expression revealed a significant correlation between TESC levels and tumor differentiation (*P* = 0.006), invasion depth (*P* = 0.025), lymph node status (*P* = 0.014), and Dukes' stage (*P* = 0.033). Furthermore, Kaplan-Meier survival curves indicated that patients with high expression of TESC were more likely to have a short overall survival than patients with low expression of TESC (*P* = 0.046, Fig. 2F), suggesting that TESC overexpression may be associated with a poor clinical prognosis. Therefore, TESC may be a potential diagnostic marker for colorectal cancer.

**Table 1:** Clinicopathologic parameters and the expression status of TESC

Characteristics	Total	TESC expression level				P-value
		High		Low/negative		
	n=143	n=77	%	n=66	%	
Age (years)						0.664
<50	26	13	50.0	13	50.0	
>50	117	64	54.7	53	45.3	
Gender						0.642
Female	68	38	55.9	30	44.1	
Male	75	39	52.0	36	48.0	
Site						0.903
Right colon	34	18	52.9	16	47.1	
Left colon	109	59	54.1	50	45.9	
Tumor size						0.285
<5 cm in diameter	61	36	59.0	25	41.0	
>5 cm in diameter	82	41	50.0	41	50.0	
Differentiation						0.006
Well	34	14	41.2	20	58.8	
Moderately	81	53	65.4	28	34.6	
Poorly	28	10	35.7	18	64.3	
Invasion Depth						0.025
T1	5	1	20.0	4	80.0	
T2	21	10	47.6	11	52.4	
T3	105	61	58.1	44	41.9	
T4	12	5	41.7	7	58.3	
Nodal status						0.014
N0	67	31	46.3	36	53.7	
N1	23	12	52.2	11	47.8	
N2	53	34	64.2	19	35.8	
Distance metastasis						0.094
M0	121	65	53.7	56	46.3	
M1	22	12	54.5	10	45.5	
Dukes' stage						0.033
A	16	6	37.5	10	62.5	
B	50	25	50.0	25	50.0	
C	55	34	61.8	21	38.2	
D	22	12	54.5	10	45.5	

TESC indicates tescalcin. Correlations between staining index scores and other categorical factors were analyzed using the Pearson chi-square test of independence.

### Increased serum TESC concentration in colorectal cancer patients

**Figure 3:**
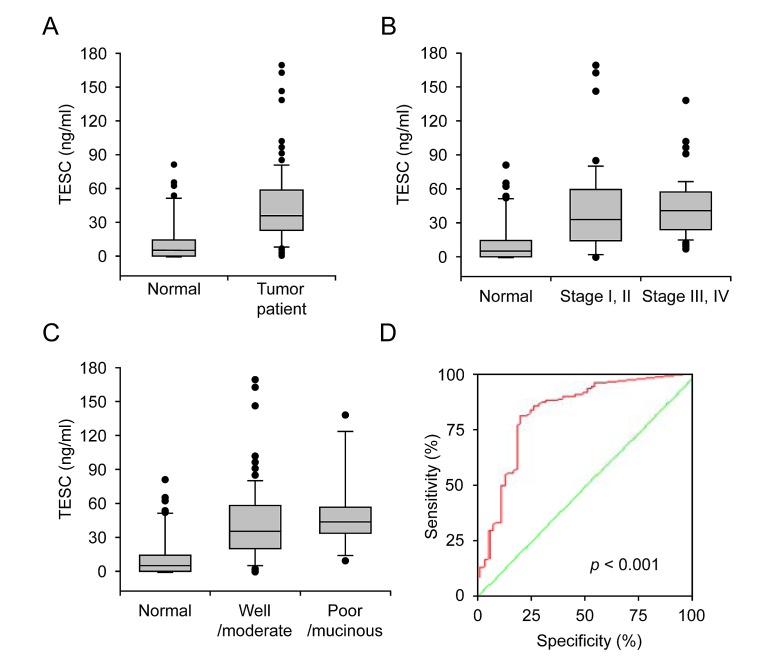
Increased serum concentration of TESC in colorectal cancer patients (A) Serum TESC concentration in control subjects (n = 54) and patients with CRC (n = 118). (B) Serum concentration of TESC in the control samples (normal), early stage colorectal cancer (stages I and II) and late stage colorectal cancer stage (stages III and IV). (C) Serum concentration of TESC in control samples (normal), well/moderately differentiated CRC, and poorly differentiated/mucinous CRC. (D) ROC curves for TESC in CRC samples. A test with perfect discrimination has a ROC plot that passes through the upper left corner (100% sensitivity, 100% specificity; green line). Therefore, the closer the ROC plot is to the upper left corner, the higher the overall accuracy of the test.

To evaluate the clinical relevance of TESC concentration as a diagnostic marker, serum specimens from colorectal cancer patients (n=118) and healthy subjects (n=54) were evaluated by sandwich ELISA. The serum concentrations of TESC in patients with CRC were significantly increased compared with those of normal individuals (42.52 ± 32.56 vs. 12.72 ± 19.88 ng/ml, respectively, *P*=6.95e-6, Fig. 3A). The serum level of TESC was increased in patients with early stage CRC (stages I and II; 41.25 ± 38.93, *P*=1.26e-5) or late stage CRC (stages III and IV; 42.94 ± 25.55, *P* = 5.09e-8) compared with control individuals (12.72 ± 19.88, Fig. 3B); however, there was no significant difference between tumor stages (stage I/II vs. III/IV; *P*=0.898). In addition, the serum TESC concentration was increased in patients with well/moderately differentiated CRC (41.32 ± 32.78, *P*=7.10e-6) and poorly differentiated//mucinous CRC (50.68 ± 32.51, *P*=2.98e-6) compared with control subjects (12.72 ± 19.88, Fig. 3C). To evaluate the sensitivity of TESC sandwich ELISA as a potential serum marker for CRC, we performed receiver operator characteristic (ROC) curve analysis of the patient samples. As shown in Figure 3D, the area under the curve for serum TESC was 0.826 for patients with CRC (95% CI, 0.753-0.899) and the cut-off point was 1.945 ng/ml. The sensitivity of the ROC curves for TESC was 94% and the specificity was 54%.

### TESC silencing attenuates CRC cell survival by inhibition of a NF-κB pathway

**Figure 4:**
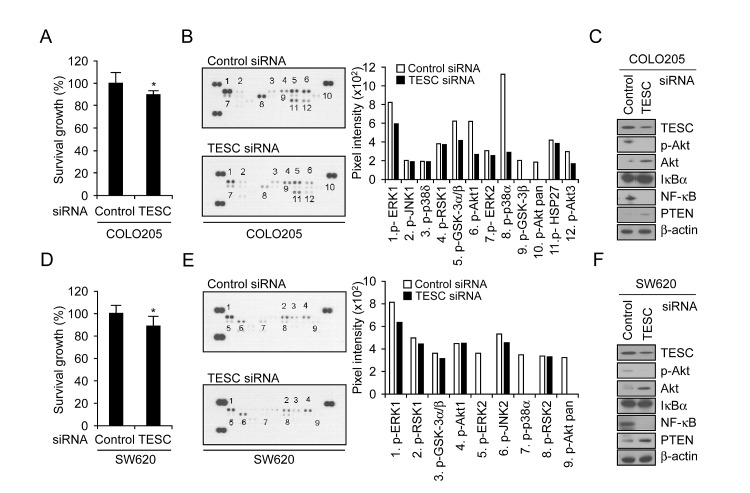
Depletion of TESC attenuates survival of colorectal cancer cells by suppression of NF-κB signaling pathway COLO205 and SW620 cells were transfected with TESC siRNA or control siRNA constructs for 2 days and subjected to the following assays: (A, D) Proliferation activity was measured by WST-1 assay; *P =0.011 and 0.019 respectively. (B, E) Phosphorylated kinase activity was measured using a phospho-MAPK array kit. (C, F) The activation of proteins involved in cell survival pathways was measured by Western blotting with antibodies against phospho-Akt, Akt, IκB, NFκB, or PTEN. Expression levels of all proteins were normalized to that of β-actin.

The data shown in Figure 1E confirm high basal expression of TESC protein and mRNA levels in COLO205 and SW620 cells. To investigate whether TESC affects progression of colorectal cancer, we analyzed proliferative activity in COLO205 and SW620 cells that were transfected with TESC siRNA. For both cell lines, the level of TESC expression in cells transfected with siRNA TESC was reduced significantly compared with cells transfected with control siRNA (Supplementary Fig. 2, Fig. 4C, F). The viability of COLO205 or SW620 cells expressing TESC siRNA was reduced by 12% or 13% respectively compared with control cells as measured by the WST-1 assay (Fig. 4A, D).

To investigate the relationship between cell viability and proliferation signaling pathways, we examined TESC-related phosphorylation of 21 mitogenic signaling effectors using the phospho-MAPK array kit. In COLO205 cells, the phosphorylation levels of ERK1, GSK α/β, p38α, Akt1, and HSP27 were decreased and phospho-GSK-3β and -pan-Akt were completely abrogated after transfection with TESC siRNA but not with control siRNA (Fig. 4B). In SW620 cells, the levels of ERK1, GSK α/β, RSK1, and JNK2 phosphorylation were decreased and phosphorylation of ERK2, p38 α/δ, and pan-Akt was completely abolished by TESC knockdown (Fig. 4E).

We confirmed the expression levels of selected proteins using Western blot analysis. Akt phosphorylation was down-regulated and NF-κB p65 activation was suppressed in TESC siRNA-expressing colorectal cancer cells (Fig. 4C, F), indicating that TESC might increase the survival of colorectal cancer cells through the Akt-dependent NF-κB pathway, but not JNK, ERK1/2, and p38 MAPK signaling pathways. We also examined proteins upstream of the Akt signaling pathway and found that expression levels of PTEN, which acts as a reciprocal PI3K suppressor in most tumor cells, were slightly increased by TESC silencing (Fig. 4C, F). These results suggest that TESC may regulate survival of colorectal cancer cells through the Akt-dependent NF-κB pathway.

### Cell proliferation in TESC-overexpressing colorectal cancer cells is enhanced through interaction between TESC and NF-κB and activation of the NF-κB promoter

**Figure 5:**
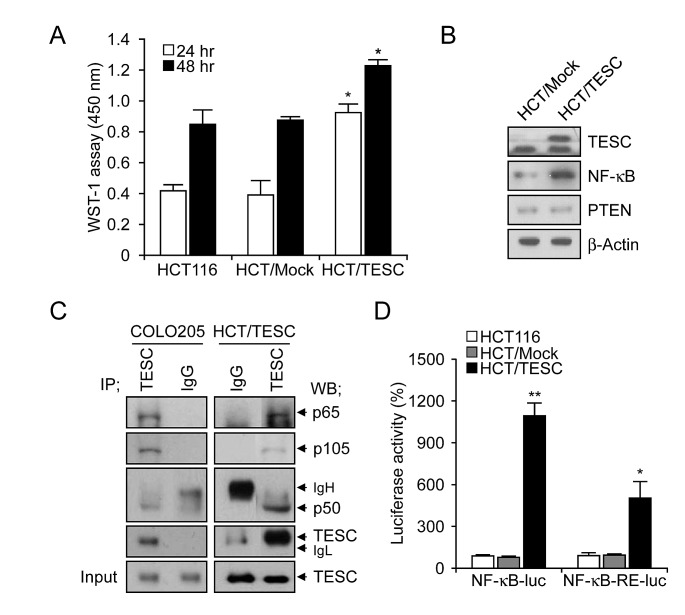
Interaction between TESC and NF-κB regulates cell proliferation in TESC-overexpressing HCT116 cells (A) Proliferation of HCT/TESC or HCT/Mock cells was measured using WST-1 reagent. *P < 0.01. (B) Western blot analysis of proteins involved in the NF-κB cell survival pathway. (C) TESC was immunoprecipitated using anti-TESC antibody, and precipitated proteins were analyzed by Western blotting using antibodies against NF-κB p65, NF-κB p50, or TESC. Total cell lysates (5% of input) were analyzed by Western blotting using anti-TESC antibody. Data are representative of three experiments. (D) NF-κB promoter activity was increased in HCT cells stably expressing NF-κB-luc and pcDNA-TESC plasmids (HCT/TESC-NF-κB-luc) compared with HCT cells expressing NF-κB-luc and pcDNA3.1 mock plasmids (HCT/Mock-NF-κB-luc), and in HCT cells stably expressing NF-κB-RE-luc and pcDNA-TESC plasmids (HCT/TESC-NF-κB-RE-luc) compared with HCT cells stably expressing NF-κB-RE-luc and pcDNA3.1 mock plasmids (HCT/Mock-NF-κB-RE-luc). Data are mean ± standard deviation from three independent experiments that were performed in triplicate. *P = 0.02; **P < 0.01.

Our previous data suggested that TESC regulates survival of CRC cells through the NF-κB pathway. To confirm these findings we generated HCT/TESC cells that constitutively expressed TESC protein and HCT/Mock control cells expressing mock vector. Cell proliferation was enhanced in HCT/TESC cells compared with HCT/Mock control cells in a time-dependent manner (Fig. 5A). Western blot analysis confirmed an increased level of TESC in HCT/TESC cells, and further showed that NF-κB expression was increased in HCT/TESC cells whereas the expression of PTEN was not significantly different from that in HCT/Mock cells (Fig. 5B). TESC was immunoprecipitated using an anti-TESC antibody, and precipitated proteins were analyzed by Western blotting using antibodies against NF-κB p65, NF-κB p50, or TESC. An interaction between TESC and NF-κB was evident in COLO205 and HCT/TESC cells (Fig. 5C), but was not detected in HCT/Mock cells (data not shown). To confirm modulation of the NF-κB promoter by TESC, the luciferase activity of NF-κB-luc or NF-κB-RE-luc plasmids transfected into HCT/TESC and HCT/Mock cells was measured 24 h after transfection. NF-κB promoter activity in HCT/TESC cells transfected with NF-κB-luc plasmid was induced up to 144% compared with transfected HCT/Mock cells. Similarly, luciferase activity driven by the NF-κB response element (RE) in the NF-κB-RE-luc plasmid was increased up to 434% in HCT/TESC cells compared with HCT/Mock cells (data not shown). In addition, we generated HCT116 cells that stably expressed NF-κB-luc or NF-κB-RE-luc plasmids and the pcDNA-TESC or pcDNA3.1 vector, respectively. The NF-κB promoter-driven luciferase activity induced by the continuous expression of TESC was significantly increased in HCT/TESC-NF-κB-luc cell lines (1166.59±90.63%, *P*<0.01) compared with HCT/Mock-NF-κB-luc cells, and was also elevated in HCT/TESC-NF-κB-RE-luc cell lines (535.39±126.02%, *P*=0.02) compared with HCT/Mock-NF-κB-RE-luc cells (Fig. 5D). These results suggest that TESC activates the NF-κB promoter to induce the NF-κB-mediated survival signal in CRC cells.

### Promotion of tumor growth by TESC in a CRC xenograft tumor model

**Figure 6:**
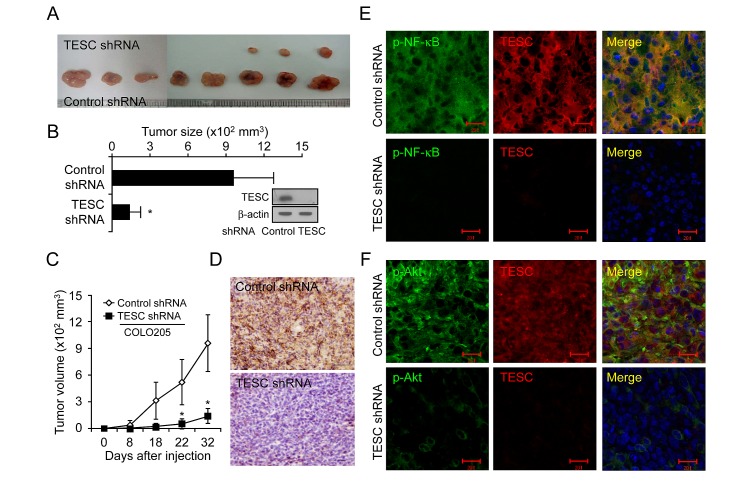
Suppression of TESC markedly reduces tumor growth in a CRC xenograft model (A-C) COLO205 cells transfected with control shRNA or TESC shRNA were inoculated into the right flank of 6-week-old nude mice. Tumor growth was monitored on the indicated days. Results represent mean tumor volume ± SD for eight animals; *P < 0.01 vs. control shRNA group. (D) Xenograft tumors formed by COLO205 cells expressing control shRNA or TESC shRNA were sectioned and stained for TESC expression. Bar = 50 μm. (E, F) Subcellular localization of TESC and phospho-NF-κB or phospho-Akt in xenograft tumors formed by COLO205 cells expressing control shRNA or TESC shRNA was detected with Alexa 555-conjugated secondary antibodies for TESC and Alexa 488-conjugated phospho-NF-κB or phospho-Akt antibodies. Bar = 20 μm.

Lastly, we investigated the effect of TESC on tumor growth *in vivo*. Tumor formation was examined after injection of CRC cells transfected with TESC shRNA or control shRNA into the flank of nude mice. Tumor growth was significantly delayed in mice injected with TESC-knockdown cells compared with mice injected with cells expressing control shRNA (Fig. 6A, C). At 32 days after injection, the average size of the tumor formed by cells expressing control shRNA was 957.9 ± 318.5 mm^3^, compared with 139.8 ± 83.5 mm^3^ for cells expressing TESC shRNA (Fig. 6B). Thus, tumor volume was suppressed up to 85.4% by TESC knockdown (*P* < 0.01). Successful knockdown of TESC by shRNA in the xenograft tumors was confirmed by Western blotting, IHC, and real-time RT-PCR (Fig. 6B, D and Supplementary Fig. 3). TESC depletion conferred a significant survival advantage compared with the control group (data not shown). These results suggest that TESC may be a potential regulator of tumor growth in CRC.

To examine the interaction between TESC and NF-κB *in vivo* in xenograft tumors, we performed immunofluorescence staining using an anti-TESC 69-18-11 mouse monoclonal antibody tagged with Alexa555 and either anti-phospho-NF-κB antibody or anti-phospho-Akt antibody tagged with Alexa488. DAPI was used as a nuclear counterstain. Immunofluorescence staining showed overexpression of TESC and phospho-NF-κB proteins and their co-localization in the cytoplasm and in the periphery of cells in xenograft tumors expressing control shRNA (Fig. 6E). In contrast, expression of TESC and phospho-NF-κB proteins was completely abolished in tumors expressing TESC shRNA (Fig. 6E). Phospho-Akt was also localized in the cytoplasm, but co-localization with TESC was not observed in xenograft tumors expressing control shRNA (Fig. 6F). Additionally, expression of TESC was completely silenced and phosphorylation of Akt was strongly inhibited in TESC-depleted xenografts. These results indicate that interaction between TESC and NF-κB may result in enhanced tumor growth of CRC.

## DISCUSSION

There is a strong need for diagnostic serum markers for colorectal cancer screening; however suitable makers have not yet been identified. In a search for possible diagnostic markers by cDNA microarray, we identified *TESC* as a gene that is over-expressed in CRC (Supplementary Table 1). TESC is a member of the calcineurin homologous protein (CHP) family and, like other family members, possesses an EF-hand calcium-binding domain. The CHP proteins regulate the activity of Na+/H+-exchange protein 1 (NHE1) and other NHE isoforms by binding to the C-terminal region of the exchange proteins [18, 21-22]. NHE1 is highly expressed in tissues that show abundant expression of TESC, such as cardiac tissues [23], and TESC and full-length NHE1 are located in the same cellular compartments, such as lamellipodia [24]. Therefore, TESC may modulate the activity, location, and degradation of NHE1 through binding to the NHE1 regulatory tail domain [25]. Although several studies have shown that NHE1 plays a role in cell proliferation, differentiation, and neoplastic transformation [26-28], the relationship between TESC and cancer progression has not previously been reported. Several lines of evidence presented in this study indicate that TESC may regulate cell growth and tumorigenicity in colorectal cancer. TESC was overexpressed in tissues and sera from patients with colorectal cancer compared with control subjects (Fig. 1, 2, and 3) and depletion of TESC using siRNA inhibited cell proliferation (Fig. 4A, D). This effect may be mediated through the modulation of cell cycle-related proteins (unpublished data) and an increase in the number of cells in G_0_ compared with control cells, as shown by FACS analysis (data not shown). Our data further indicate that these effects of TESC were mediated via the downregulation of NF-κB. Silencing of TESC suppressed NF-κB expression in COLO205 or SW620 colorectal cancer cells (Fig. 4C, F), but did not affect PTEN expression (Fig. 4C, F). Thus, TESC may regulate NF-B expression but not NF-κB upstream signaling pathways such as PTEN, and TESC knockdown may suppress the proliferative activity of colorectal cancer cells by inhibiting NF-κB pathway signaling. Several tumor cell growth factors are cytokines that are modulated by NF-κB [29]. For example, the receptors for IL-1 and tumor necrosis factor (TNF) are themselves regulated by NF-κB and also modulate cell proliferation through NF-κB [30]. Moreover, attenuation of NF-κB activity inhibits liver metastasis [31].

The Akt signaling pathway is also involved in the regulation of cell proliferation, survival, and motility, and there is evidence of cross-talk between the Akt and NF-κB signaling pathways [32]. Akt is a serine/threonine protein kinase that is activated by phosphorylation and regulates cell proliferation and growth, and the migration and invasion of cancer cells, thus promoting tumorigenesis [31, 33-35]. As shown in Figure 4B, C, E and F, Akt phosphorylation was attenuated in TESC-depleted COLO205 or SW620 cells compared with cells transfected with control siRNA. Therefore, TESC may mediate the survival and proliferation of colorectal cancer cells by promoting the activation of NF-κB and Akt.

The role of TESC in colorectal tumor growth was confirmed in vivo by the strong suppression of tumor growth in xenograft tumors expressing TESC shRNA compared with tumors expressing control shRNA (Fig. 6A-C). Immunofluorescence staining showed overexpression of TESC and the phospho-NF-κB protein and their co-localization in the tumor cell cytoplasm and periphery in control xenograft tumors (Fig. 6E). However, although phospho-Akt was also localized in the cytoplasm, it did not show co-localization with TESC (Fig. 6F). On the basis of these results, we suggest that TESC interacts with NF-κB and thus enhances tumorigenesis in CRC.

Silencing of TESC also significantly attenuated the phosphorylation of glycogen synthase kinase 3 (GSK3) protein isoforms GSK3α and GSKβ (Fig. 4) and increased levels of E-cadherin expression (unpublished data) in TESC-depleted colorectal cancer cells. Glycogen synthase kinase 3 (GSK3) is a serine/threonine kinase that is known to be associated with metastasis [36]. β-Catenin plays a role in cell-cell adhesion and transcription and is destabilized by phosphorylation by GSK3 [37-38]. During various stages of cell migration, the actin cytoskeleton, microtubules, and adhesion turnover are modulated by GSK3 [38]. We hypothesize that expression of TESC may regulate cell migration and invasion through regulation of NF-κB, Akt, and GSK3. Work is ongoing to elucidate the detailed molecular mechanisms underlying the role of TESC in metastatic processes of colorectal cancer.

In conclusion, this study reveals an important role of TESC in the progression of colorectal cancer through its interaction with NF-κB. TESC may be a potential oncotarget in colorectal cancer and may have potential clinical value as a diagnostic marker for colorectal cancer screening.

## MATERIALS AND METHODS

### Illumina microarray and reverse transcription-polymerase chain reaction

cDNA microarray analysis was performed on 66 paired samples of CRC tissue and adjacent normal mucosa provided by Samsung Medical Center (Seoul, Korea). Total RNA was isolated using an RNeasy midi-kit (Qiagen, Hilden, Germany) following the manufacturer's instructions. cDNA microarrays (48K, Human-6 V2) were purchased from Illumina Inc. (San Diego, CA) [39].

For RT-PCR, total RNA was extracted from CRC cell lines and normal/tumor paired tissues using TRIzol reagent (Invitrogen, Carlsbad, CA) according to the manufacturer's instructions. After quantification, 5 μg of RNA was annealed to oligo (dT) at 65°C for 5 min and the RNA-oligo (dT) mixtures were incubated with reverse transcriptase and dNTPs at 42°C for 1 hr using the ProSTAR First-Strand RT-PCR kit (Stratagene, CA). cDNA was used as a template for PCR with Ex Taq polymerase (Takara, Japan). The specific primers used for PCR were as follows: TECS, 5'-CCT ACC ATT CGC AAG GAG AA-3' (sense) and 5'-TTC TCG ATG TGA GGG TTT CC-3' (antisense); β-actin, 5'-AAG GCC AAC CGC GAG AAG AT-3' (sense) and 5'-TGA TGA CCT GGC CGT CAG G-3' (antisense). The optimized PCR conditions were as follows: 1 cycle of 94°C for 5 min; 35 cycles of 94°C for 40 sec, 57°C for 40 sec, and 72°C for 30 sec; and final extension at 72°C for 7 min. Relative levels of gene expression were normalized to GAPDH expression.

### Tissue samples and immunohistochemistry

Tissue samples were obtained from patients who underwent routine surgery for colorectal cancer between January 2000 and June 2005 at the Department of Surgery, Eulji University Hospital (Daejeon, Korea). All patients understood the donation procedure and signed a written informed consent that was approved by the institution's ethics committee [40]. For immunohistochemical analysis, tissue samples were fixed in 10% neutral buffered formalin and embedded in paraffin wax. After serial sectioning at 4-μm thickness, the tissue slices were mounted on charged Superfrost Plus glass slides (Fisher Scientific, Rochester, NY). Anti-TESC antibody (1:200 dilution; Catalog number 11125-1-AP, ProteinTech, Chicago, IL) was used for staining as described in our previous paper [40].

### Sandwich ELISA of serum samples and ROC curve

Serum samples of 118 CRC patients and 54 healthy donors were provided by the Samsung Medical Center after receipt of written informed consent from all patients. Sera were allowed to clot and were stored at -70°C. Serum concentration of TESC in CRC patients was determined by sandwich enzyme-linked immunosorbent assay (ELISA). Experimental procedures and statistical analysis by ROC curve are described in Ji et al (2010) [39].

### Cell lines and culture conditions

COLO205, SW620, and HCT-116 colorectal cancer cell lines were purchased from American Type Culture Collection (Rockville, MD) and cultured in DMEM supplemented with 100 U/ml penicillin, 100 μg/ml streptomycin, 25 ng/ml amphotericin B, and 10% fetal bovine serum (FBS) (GIBCO, Grand island, NY) at 37°C in a humidified incubator with 5% CO_2_. Human TESC cDNA (NM_017899) was amplified from a cDNA library and inserted into the EcoRI/HindIII site of pcDNA3.1 vector (Invitrogen). HCT116 cells were transfected with pcDNA3.1 mock vector or pcDNA3.1 containing the TESC coding region using Lipofectamine 2000 (Invitrogen) as recommended by the manufacturer [41] to give the established cell lines HCT/Mock and HCT/TESC, respectively.

### WST-1 assay and phospho-MAPK array

COLO205 and SW620 colorectal cancer cells were transfected with 100 nM control siRNA or TESC siRNA and subjected to survival analysis using the WST-1 assay (Boehringer Mannheim, Mannheim, Germany) according to the manufacturer's protocol. Relative levels of phosphorylated mitogen-activated protein kinases (MAPKs) and other serine/threonine kinases were determined using a phospho-MAPK array from R&D systems, Inc. (Minneapolis, MN) according to the manufacturer's protocol [41].

### Immunoprecipitation and western blot analysis

Cell lysates were reacted with TESC antibody or control IgG for 4 h at 4°C and the complexes between antigen and antibody were precipitated with protein G-conjugated agarose (Roche, Basel, Switzerland) by overnight incubation at 4°C. The immunoprecipitated complexes were cleared and analyzed by western blot analysis as we described previously [41].

### siRNA transfection and luciferase reporter assay

TESC siRNA and control siRNA were purchased from Samchully Pharm. Co. (Seoul, Korea). The primer sequences of TESC siRNA were sense 5'-GCU UCU CAU CGG AUC AGA UTT-3' and antisense 5'-AUC UGA UCC GAU GAG AAG CTT-3'. Colorectal cancer cells were transfected with TESC siRNA or control siRNA using Lipofectamine Plus (Invitrogen) according to the manufacturer's protocol. The luciferase reporter assay was performed using a NF-κB promoter-luciferase reporter plasmid, NF-κB-luc, provided by Dr. Kim JW (KRIBB, Daejeon, Korea) and pGL4.32 (luc2P/NF-κB-RE/Hygro) vector, termed NF-κB-RE-luc, purchased from Promega (Madison, WI). HCT116, HCT/Mock, and HCT/TESC cells were transfected with NF-κB promoter luciferase plasmid, NF-κB-luc, and NF-κB-RE-luc using Lipofectamine 2000 as recommended by the manufacturer [41].

### Nude mouse xenograft model of colorectal cancer

Six-week-old nude mice were purchased from Charles River Laboratories and kept under sterile specific pathogen-free conditions. All experiments were performed following the Animal Care and Use guidelines of the Korea Research Institute of Bioscience and Biotechnology (Daejeon, Korea). For the xenograft assay, COLO205 cells expressing TESC-targeting shRNA (sc-96026-V, Santa Cruz Biotechnology) or control shRNA (sc-108080) were harvested, cleared twice in phosphate-buffered saline (PBS), and 1 × 10^6^ cells resuspended in 0.1 ml of PBS were injected *s.c.* into nude mice (n=8 for each group). Tumor growth was assessed by measuring tumor length and width using a caliper at 3- or 4-day intervals and tumor volume was calculated using the formula: Volume = 0.523*Lw*^2^ (*L* = length, *w* = width). The percentage of surviving mice was calculated by recording events related to tumor growth (tumor size, >2,000 mm^3^) over 50 days.

### Immunostaining and laser-scanning confocal microscope analysis

TESC immunoreactivity was analyzed using a semiquantitative scoring system that included both the staining intensity and the percentage of positively stained neoplastic cells percentage as described by Kim et al (2011) [40]. CRC xenograft tumor tissues were fixed in 4% paraformaldehyde and then incubated in 30% sucrose buffer at 4°C overnight. Free-floating transverse frozen sections of 30 μm thickness were cut in a cryostat and blocked with 5% goat serum in 0.2% Triton X-100 for 1 h at room temperature. The sections were incubated at 4C overnight with a mouse anti-TESC69-18-11 monoclonal antibody (1:1000, KCTC11790BP, Korean Collection for Type Cultures, KRIBB). The procedures for immunofluorescence staining and laser-scanning confocal microscope analysis have been described previously [42].

### Statistical analysis

Data are shown as mean ± standard deviation and significance of statistical analysis was assessed using two-tailed, unpaired Student *t* tests. *P*-values <0.05 were considered significant. Chi-square or Fisher's exact tests were used to assess the univariate associations of baseline characteristics. Survival was assessed using the Kaplan–Meier method. All analyses were performed using the SPSS program (Ver. 19; IBM).

## SUPPLEMENTARY FIGURES AND TABLES


