# Supplementary material for: The EF-hand calcium-binding protein tescalcin is a potential oncotarget in colorectal cancer
Source: Oncotarget. 2014 Mar 19;5(8):2149–60. doi: 10.18632/oncotarget.1851 (PMC4039152; doi:10.18632/oncotarget.1851)
Supplement: Supplementary file 1 [file oncotarget-05-2149-s001.pdf]

The EF-hand calcium-binding protein tescalcin is a potential oncotarget in colorectal cancer

- Kang *et al* -

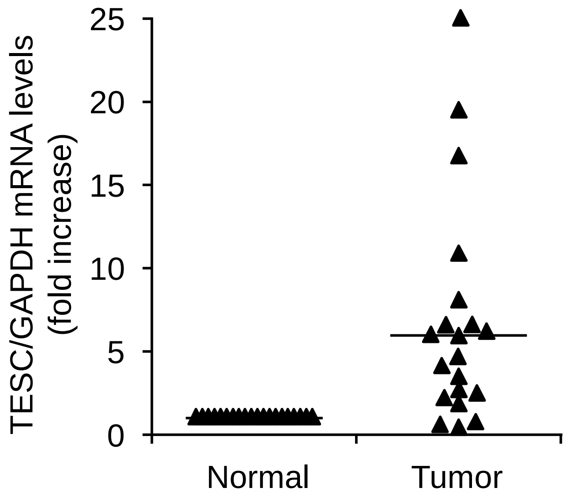

Supplementary Figure 1: TESC mRNA and protein expression in human colorectal tissues was analyzed by real time RT-PCR and western blot analysis respectively. The expression level of TESC in each sample was determined as the ratio of TESC to GAPDH. The expression of tumor TESC was given as a relative value compared with TESC/GAPDH of matched normal controls (n=20; taken as 1.0).

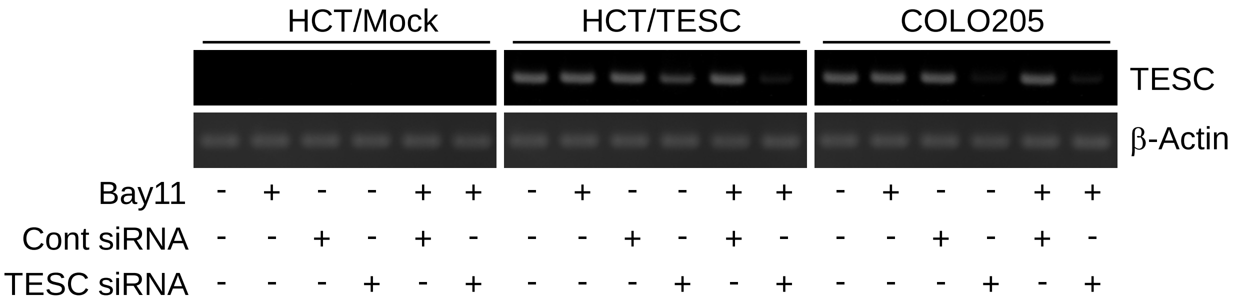

Supplementary Figure 2: Effect of combination treatment with NF-κB inhibitor and TESC siRNA on TESC expression of human colorectal cancer cells HCT/Mock, HCT/TESC, and COLO205. Cells were treated with the NF-κB inhibitor Bay 11-7082 (10 μM) under the transfection with TESC siRNA. The transcriptional expression of TESC was measured by RT-PCR analysis. β-Actin was used as the loading control. Data are representative of two experiments.

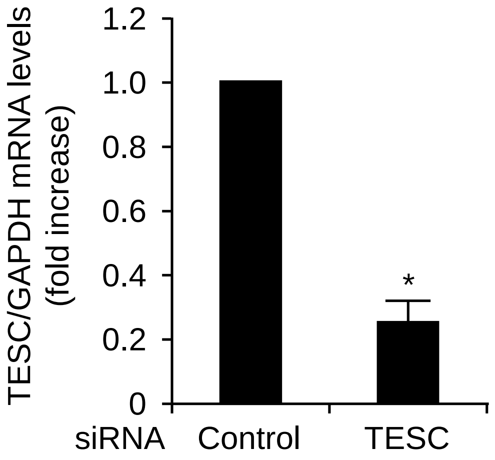

Supplementary Figure 3 - Real-time RT-PCR analysis of TESC expression in xenograft tumors formed by COLO205 cells expressing control shRNA or TESC shRNA. The level of expression of TESC in each sample was determined as the ratio of TESC to GAPDH. The expression of TESC in TESC shRNA-expressing xenograft tumor was calculated as a relative value of TESC/GAPDH compared with TESC/GAPDH of control shRNA-xenograft tumor (taken as 1.0). $*P < 0.05$. Data are representative of two experiments.

Supplementary Table 1.

Differentially expressed genes between tumor and non-tumor tissues of patients with colorectal cancer

Gene Symbol	Gene Title	Regulation (tumor vs normal	Fold change-average	Patient's number of 2-fold change
COL7A1	collagen, type VII, alpha 1	up	2.007	58
SNTB1	syntrophin, beta 1 (dystrophin-associated protein A1, 59kDa, basic com	up	1.288	50
UTP11L	UTP11-like, U3 small nucleolar ribonucleoprotein	up	1.258	51
PI3	peptidase inhibitor 3, skin-derived	up	1.153	51
IL1B	interleukin 1, beta	up	1.9	55
MMP12	matrix metallopeptidase 12	up	1.743	53
SULT2B1	sulfotransferase family, cytosolic, 2B, member 1	up	1.691	59
SLCO4A1	solute carrier organic anion transporter family, member 4A1	up	2.89	63
TESC	tescalcin	up	2.41	57
KPNA2	karyopherin alpha 2 (RAG cohort 1, importin alpha 1)	up	2.27	66
MTHFD1L	methylenetetrahydrofolate dehydrogenase (NADP+ dependent) 1-like	up	2.19	66
UBE2C	ubiquitin-conjugating enzyme E2C	up	2.17	63
SRPX2	sushi-repeat containing protein, X-linked 2	up	2.16	50
NKD2	naked cuticle homolog 2	up	2.15	55
NFE2L3	nuclear factor, erythroid 2-like 3	up	2.291	65
ESM1	endothelial cell-specific molecule 1	up	5.7	65
MMP1	matrix metallopeptidase 1 (interstitial collagenase)	up	3.11	50
AZGP1	alpha-2-glycoprotein 1, zinc-binding	up	3	51
EGFL6	EGF-like-domain, multiple 6	up	2.97	51
COL11A1	collagen, type XI, alpha 1	up	2.9	55
CXCL6	chemokine (C-X-C motif) ligand 6	up	2.67	53
COL1A1	collagen, type I, alpha 1	up	2.4	45
AGT	angiotensinogen (serpin peptidase inhibitor, clade A, member 8)	up	2.38	44
TCN1	transcobalamin I (vitamin B12 binding protein, R binder family)	up	3.46	46
CEL	carboxyl ester lipase	up	3.35	48
GDF15	growth differentiation factor 15	up	2.87	40
ALDOB	aldolase B, fructose-bisphosphate	up	2.53	42

Supplementary Table 2.

Expression of TESC in CRC tissues by real-time RT-PCR

	N	Mean±SD	*p* value
Tumor size (cm)			
<5 cm in diameter	2	2.48±3.09	
≥5 cm in diameter	8	12.35±20.15	0.26
Not defined	10		
Differentiation			
Well	3	94.31±85.45	0.008
Moderately	15	11.46±18.25	
Not defined	2		
Invasion Depth			
T2	2	9.91±13.45	
T3	15	27.19±48.80	0.25
T4	1	6.59	
Not defined	2		
Nodal status			
N0	11	35.2±55.58	
Not defined	9		
Distance metastasis			
M0	9	5.00±5.81	
M1	4	34.19±38.70	0.04
Not defined	7		
